# Sea level rise and coastal flooding risks in the Gulf of Guinea

**DOI:** 10.1038/s41598-024-80748-w

**Published:** 2024-11-28

**Authors:** Franck Eitel Kemgang Ghomsi, Björn Nyberg, Roshin P. Raj, Antonio Bonaduce, Babatunde J. Abiodun, Ola M. Johannessen

**Affiliations:** 1 Deutsches Geodätisches Forschungsinstitut, Technische Universität München (DGFI-TUM), Munich, Germany; 2 Nansen-Tutu Centre for Marine Environmental Research, Department Oceanography, University of Cape Town, Cape Town, South Africa; 3 Geodesy Research Laboratory, National Institute of Cartography, P.O. Box 157, Yaoundé, Cameroon; 4 Analytics, Innovation District Solheimsviken 7c, 5054 Bergen, Norway; 5 Nansen Environmental and Remote Sensing Center and Bjerknes Center for Climate Research, Bergen, Norway; 6 Climate System Analysis Group, Department of Environmental and Geographical Science, University of Cape Town, Cape Town, South Africa; 7 Nansen Scientific Society, Bergen, Norway

**Keywords:** Gulf of Guinea, Sea level rise, Coastal flooding, CMIP6 projections, Sustainable development goals, Environmental impact, Climate-change impacts, Climate-change mitigation, Projection and prediction

## Abstract

The Gulf of Guinea (GoG) is highly vulnerable to sea level rise, with projections indicating a significant increase in permanently inundated land by 2100, ranging from 1,458.1 to 4,331.7 km^2^. This study evaluates the severity of potential coastal inundation in the GoG by comparing sea level rise projections from eight reliable CMIP6 models with historical sea surface height (SSH) data from 1993 to 2015 and current onshore topography. Eight model simulations were selected based on their accuracy in reproducing sea level variability in the Tropical Atlantic and the GoG, and their consistency in reflecting the one-month connection lag between equatorial-driven waves and Kelvin Coastal Trapped Waves (CTWs) along the GoG, critical for predicting regional ocean dynamics. Our findings indicate that this connection lag will remain consistent over time. Under high-emission scenarios, up to 95% of coastal areas could be inundated, potentially displacing 2 million people posing a socio-economic shock, given the region’s low GDP and heavy reliance on fisheries. The loss of cultural heritage and livelihoods further compounds the challenges. These findings emphasize the urgent need for targeted adaptation strategies and robust early warning systems, in line with the UN’s Sustainable Development Goals (SDGs), particularly SDG 13 (Climate Action) and SDG 14 (Life Below Water). This study offers a precise and regionally relevant assessment of future risks, providing a foundation for informed policy interventions to mitigate the impacts of climate change and protect vulnerable communities in the GoG.

**Supplementary Information:**

The online version contains supplementary material available at 10.1038/s41598-024-80748-w.

## Introduction

**Fig. 1:**
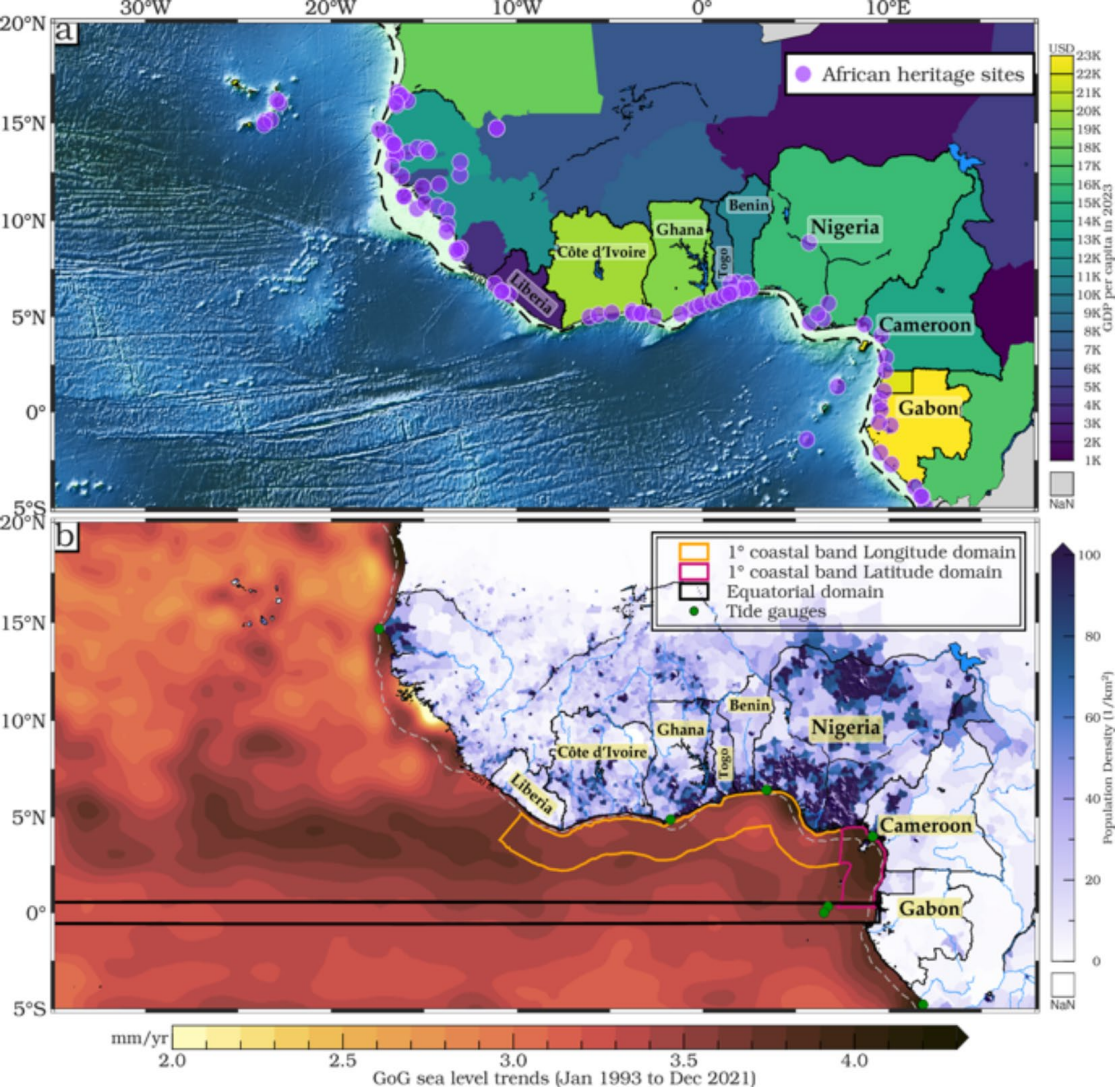
(**a**) GDP per capita in 2023 across the GoG (IMF, 2024), with African heritage sites displayed in purple and the 200 m isobath depicted in dashed black line. (**b**) Sea level trends (mm/yr) in the GoG from 1993 to 2021, computed from gridded monthly SLAs at 1/4-degree spatial resolution distributed by Copernicus Marine Environment Monitoring Service (CMEMS). The mainland population density distribution in 2021 (people/km^2^), based on data from Grid Population of the World (GPW, https://sedac.ciesin.columbia.edu/data/collection/gpw-v4), is superimposed on the geographical boundaries of the equator (black, 10° E-50° W and 1° N-1° S) and the GoG coastal areas of interest: Latitudinal range (0–4° N, 1° wide coastal band) outlined in pink and longitudinal range (10° E-10° W, 1° wide coastal band) outlined in orange. The 200 m isobath is indicated by the white dashed line along the coast. All plots were generated using Generic Mapping Tools (GMT), Version 6.5.0 (https://www.generic-mapping-tools.org/).

The Global Mean Sea Level (GMSL) rose by approximately 11.1 cm from 1993 to the end of 2023^1,2^, with the rate of increase more than doubling over this period. Starting at around 2.1 ± 1.0 mm/yr in 1993, the rate accelerated to 4.5 ± 1.0 mm/yr by the end of 2023, well above the 20th-century average of 1.7 mm/yr. This accelerated rise is driven primarily by thermal expansion of ocean waters, melting glaciers and ice sheets, and changes in land water storage, largely resulting from rising global temperatures. Future estimates suggest an acceleration of this trend, potentially resulting in a rise of over 2 m by 2100, posing an unprecedented risk to coastal regions globally. Low-lying coastal and estuarine regions, particularly the Gulf of Guinea (GoG)^3,4^, which stretches from Liberia to Gabon (Fig. 1) and possibly home to around a billion people by 2030, face serious threats from rising sea levels, coastal land subsidence^5,6^ and extreme events^7–9^. GoG is already experiencing a sea level rise at a rate of 3.89 mm/yr (Fig. 1) along the coast^10,11^, which corresponds to a mean sea level rise of about 11.3 cm over the period 1993–2021, approximately 10.51% higher than the global average rate (3.52 mm/yr). In the GoG, sea level rise leads to coastal erosion, and shoreline modifications and saltwater intrusion resulting in salinization of surface and groundwater^12–14^. These changes increase the frequency and intensity of coastal flooding, degrade, and destroy habitats and ecosystems like coral reefs and islands, and reshape shorelines^15,16^, directly impacting biodiversity^17^. Critical coastal infrastructure, urban areas, and the tourism industry which includes heritage sites from historical slavery time-period, commercial ports, and trade routes are at significant risk^18,19^. These threats drive human migration, jeopardize the livelihoods and safety of vulnerable coastal communities, endanger cultural heritage sites, and undermine renewable energy projects. Kulp and Strauss^20^ highlights the potential displacement of millions due to sea level rise globally, and similar risks apply to the densely populated GoG region, underscoring the need for adaptive solutions specific to the area’s socio-economic context.

Economically, the GoG is heavily reliant on marine resources, facing substantial challenges with an average Gross Domestic Product (GDP) of $739 billion^21,22^, placing these nations among the lowest globally (Fig. 1). Rising sea levels pose a critical threat to coastal communities and their livelihoods, yet efforts to mitigate these impacts have been insufficient^23^. Adaptive approaches are limited, particularly with regard to human-made flood defenses and community-driven adaptation strategies^24^. This deficit places further strain on GoG communities and ecosystems already under threat from climate change. The GoG’s vulnerability is compounded by inadequate strategies to protect its coastal infrastructure^25^, fragile ecosystems, and the millions of people who depend on them, especially the youth, who are projected to make up a quarter of Africa’s population by 2050^26^. Additionally, the GoG faces significant limitations in monitoring infrastructure, with a scarcity of tide gauges providing long-term data^27,28^. This lack of reliable observational data hinders accurate regional forecasting and early warning systems, putting GoG local communities, especially those reliant on fishing and coastal resources, at heightened risk. Consequently, comprehensive assessments that capture both physical and socio-economic vulnerabilities are crucial to understanding and mitigating risks in the GoG. Immediate and coordinated action is essential to safeguard GoG from the severe impacts of climate change and to ensure the sustainability of its maritime economy. Progress towards the Sustainable Development Goals (SDGs)^29^ is crucial in this context. The SDGs represent the UN’s pledge to "leave no one behind," asserting that "no goal or target shall be considered achieved unless it is met for all social and economic groups". However, the scarcity and unreliability of tide gauges in the GoG, due to their short records, hinder effective monitoring and early warning of sea level rise^10,11,30,31^. This knowledge gap exacerbates the struggles of local communities, especially those dependent on fishing and cartographic expansion, who already feel marginalized in the face of growing insecurity.

The interconnectedness of SDG 14 (Life below water) with other goals—such as SDG 1 (No poverty), SDG 2 (Zero hunger), SDG 3 (Good health and well-being), SDG 4 (Quality education), and SDG 8 (Decent work and economic growth)—highlights the urgent need to address sea level projections and develop innovative early warning systems. Accurate sea level projections are critical for the GoG to mitigate the adverse effects of sea level rise, such as coastal flooding and ecosystem degradation. Addressing these challenges is crucial for the GoG to advance towards these SDGs and ensure the well-being and prosperity of its inhabitants and humanity’s future.

This study aims to assist in achieving the UN SDGs by comprehensively evaluating sea surface height (SSH) data over the GoG and project future changes using CMIP6 models and historical data. By applying both SSH data and multi-model comparisons, this study addresses gaps in localized projections, as previously emphasized by Jackson and Jevrejeva^17^ in regional sea-level analyses.

By aligning with SDG 13 (Climate Action) and SDG 14 (Life Below Water), the study seeks to enhance regional resilience to climate change impacts. Firstly, detrended interannual SSH data (1993–2015) are compared with CMIP6 model outputs over the same period to identify the models that most accurately replicate regional climate dynamics. Then, based on the selected models, an ensemble mean is generated to project various climate scenarios up to the next century. This ensemble approach, as supported by Slangen, et al.^32^, enhances the reliability of projections by reducing uncertainties inherent to individual model simulations for understanding the potential impacts of sea level rise. Finally, these projected scenarios are utilized to create inundation maps, providing critical insights into potential future climate impacts, and informing adaptation and mitigation strategies. These assessments aim to support planning activities for effective adaptation to future sea level rise over the Tropical Atlantic, in particular the GoG, while contributing to the broader SDG objectives of fostering well-being, sustainability, and resilience in coastal regions worldwide.

## Results

### Historical scenario validation

We evaluated historical data changes of sea surface height (SSH) across the Tropical Atlantic Basin using the most recent CMIP6 multi-model ensemble simulations to support planning activities for effective adaptation to future sea level rise^33^. Among the 28 CMIP6 models evaluated from historical data (Fig. S1 Supplementary material) only eight of them were able to reproduce the (interannual and seasonal) variability in the Tropical Atlantic, in particular along the three selected domains stretching from the equatorial band to the GoG (Table 1).

**Table 1:** List of 8 CMIP6 models selected based on best performing models in the region.

Model name	References	Correlation of detrended CMIP6 interannual SSH with observations
CNRM-CM6-1	Voldoire, et al.^34^	0.79
EC-Earth3-Veg	Döscher, et al.^35^	0.91
HadGEM3-GC31-LL	Roberts, et al.^36^	0.60
IPSL-CM6A-LR	Boucher, et al.^37^	0.88
MIROC6	Tatebe, et al.^38^	0.93
MRI-ESM2-0	Yukimoto, et al.^39^	0.80
NESM3	Cao, et al.^40^	0.89
NorESM2-MM	Seland, et al.^41^	0.94

**Fig. 2:**
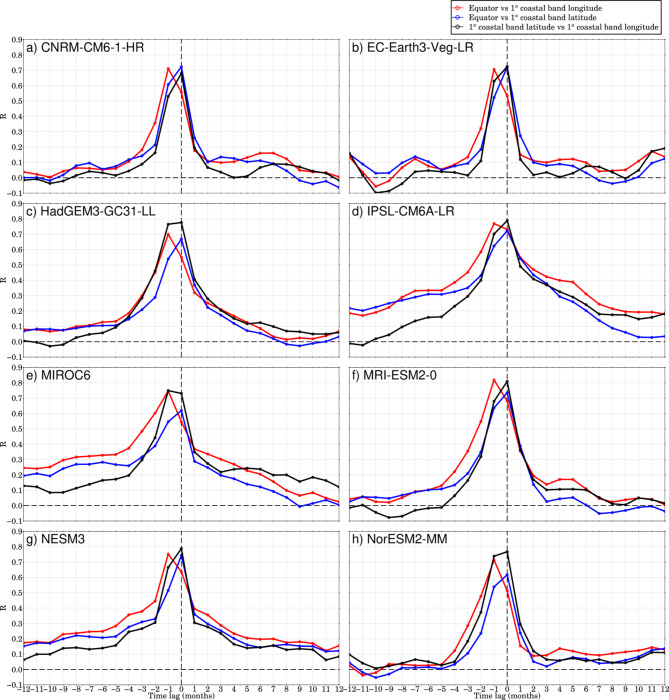
Cross-correlation between monthly SSH in the equatorial band and the 1° coastal band in longitude (blue), between the equatorial band and the 1° coastal band in latitude (red), and between the 1° coastal band in latitude and longitude (black) with reference to Fig. 1b. The analysis covers the period from 1993 to 2021, focusing on the propagation of waves from the open ocean into the coastal region of the GoG. Correlations that are statistically significant at the 95% confidence level are indicated by dots.

**Fig. 3:**
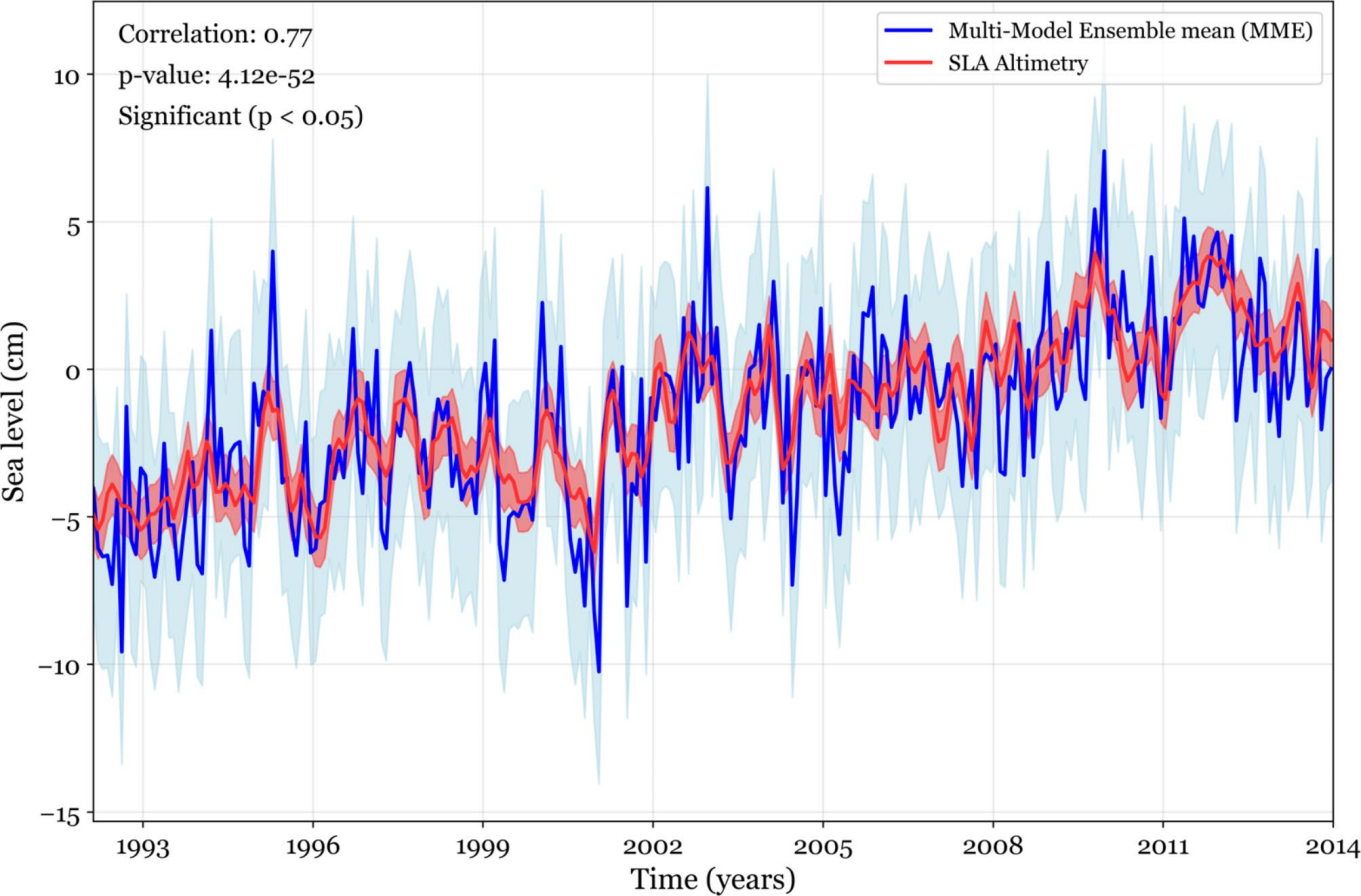
Sea level time series of altimetry-based and multi-model ensemble mean (MME) from historical simulations of eight models, with shaded areas representing their respective standard deviations.

Figure 2 demonstrates the cross-correlation between three selected areas, highlighting the ocean variability connection that links the equatorial Atlantic with the GoG coastal regions. Our primary finding is the consistent one-month lag between the equatorial band and the GoG longitude band^10^. This stability makes it an effective predictor of the variability in Kelvin Coastal Trapped Waves (CTWs) propagation. The sea-level signals in the climate models exhibit a cross-correlation similar to that observed in Ghomsi, et al.^10^. As the magnitude of the correlations increases, the shape of the cross-correlation function remains unchanged, confirming the validity of the selected models. Notably, while the one-month connection lag between equatorial-driven waves and GoG CTWs remains unchanged in historical data, the cross-correlation with a positive correlation extends beyond 10 months, which is longer than observed. To further substantiate these findings, we have found a significant correlation of 0.77 between the observed sea-level rise and the multi-model ensemble mean (MME) of the historical simulations from the eight selected models (Fig. 3). This high correlation not only validates the model outputs but also suggests that the MME provides a reliable representation of historical sea-level trends in the GoG. The comparison of observed and simulated sea-level rise rates enhances our understanding of the dynamics at play and supports the robustness of the model in predicting future sea-level scenarios.

###  Long-term projected sea-level rise and coastal inundation

**Fig. 4:**
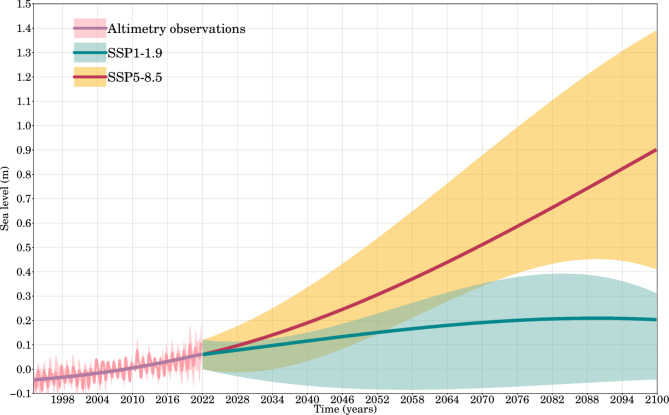
GoG altimetry-based sea level (1993–2021) and SSP1-1.9 (very low-emission) and SSP5-8.5 (very high-emission) sea level rise scenarios (2022–2100) obtained from 8 scenarios. The shaded areas represent the probable ranges for the observation-based and ensemble mean scenarios, while the solid lines represent the median values.

Figure 4 shows the projected value of sea level rise in the GoG until 2100, with a likely (5th–95th percentile) range between 0.20 and 1.38 m. This overlaps with observation-based extrapolations, ranging from 0.25 to 0.58 m for the low scenario (SSP-1.9) and 0.63–1.01 m for the high scenario (SSP5-8.5), consistent with estimates from Hall et al.^42^, Hall et al.^43^ the IPCC Sixth Assessment Report (AR6), and Slangen, et al.^44^. While the numerical ranges suggest proximity (0.33 m vs. 0.38 m), the broader uncertainty and potential outcomes associated with the SSP5-8.5 scenario contribute to a larger shaded area in Fig. 4, reflecting greater variability in projections due to higher emissions.

**Fig. 5:**
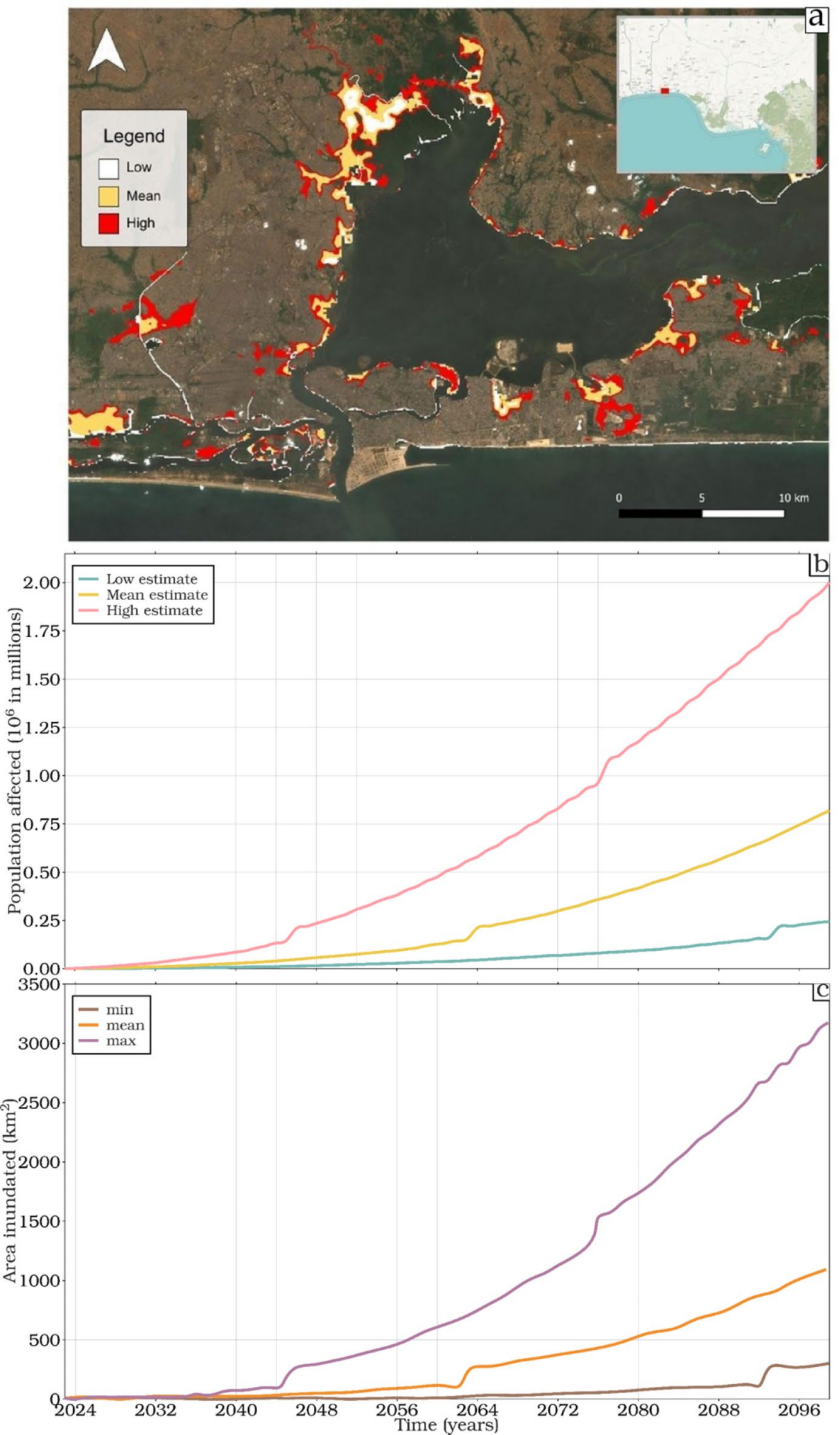
(**a**) Examples of coastal inundation extracted from the Flood Tool’s (https://bjornburrnyberg.users.earthengine.app/view/slr-gulf-of-guinea) built extent for Lagos, Nigeria based on low, mean or high projected sea-level rise levels for the SSP5-8.5 scenario at the year 2100. Sentinel-2 imagery background imagery is courtesy of the European Space Agency (ESA), and an overview map is based on OpenStreetMap © contributors under the Open Database License (ODbL). Maps were produced using QGIS^47^. Projected impacts of sea level rise in the GoG are further illustrated in (b) the population affected based on the WorldPop Project^46^ and (c) the area inundated, under the SSP5-8.5 scenario at the year 2100.

The long-term projected sea level rise scenarios depicted in Fig. 5a provides a stark warning for coastal regions along the GoG, particularly in areas like Lagos, Nigeria. Here we assess the potential extent of coastal inundation by comparing rising sea levels against present-day onshore topography^45^ and evaluating the human impact using 2020 population distribution data^46^.

Figure 5b highlights the projected population affected under the SSP5-8.5 (very high-emission) scenario from 2022 to 2100 given the same population density as reported in 2020. Significant populations at risk, especially in the high estimates scenarios, signal the potential for widespread displacement and profound socio-economic disruptions in the region. By 2100, the high estimate reaches approximately 2 million people, approximately 800 thousand people by a mean estimate and 250 thousand people by a low estimate. These results show the urgent need for proactive and adaptive strategies to mitigate these risks.

The consequences extend beyond population displacement. Figure 5c illustrates the projected area of land inundated under the same scenario. Similar to the trends in population displacement, the maximum estimate for land inundation shows notable increases around 2045 and 2076, threatening significant land loss, including vital coastal infrastructure and ecosystems. By 2100, the maximum estimate exceeds 3500 km2, posing a severe threat not only to critical habitats and biodiversity but also to the region’s economic stability, food security, and cultural identity.

The loss of such a significant portion of coastal area could devastate the socio-economic fabric of the GoG. Many of these coastal regions are home to dense populations, where livelihoods are closely tied to the land and the sea. The inundation of agricultural lands, fishing areas, and urban centers would lead to food insecurity, loss of income, and increased poverty, disproportionately affecting the most vulnerable communities. Additionally, the potential submersion of culturally and historically significant sites threatens the erasure of important aspects of the region’s heritage, further compounding the social impact of such environmental changes.

The synchronized jumps in both population displacement and land inundation in 2045, 2062, 2076, and 2093 suggest periods of increased risk, likely driven by a combined impact of sea level rise and the shoreline topography. These specific years are critical as they may correspond to thresholds or tipping points within climate models or regional trends, marking moments where the effects of sea level rise become particularly pronounced. Even the low estimate and minimum values show a notable increase in 2093, indicating significant impacts under more conservative projections. Likely such spikes indicate critical stages whereby sea level rise in the region will flood a larger protected area behind a natural or man-made barrier. Once breached, it will have a major impact on the infrastructure, environment, and human livelihoods within a relatively short space of time. These catastrophic risks will be different from the gradual steady increase witnessed in other decades, both of which will require different responses. The findings highlight the dual challenges of human displacement and environmental degradation due to sea level rise, necessitating integrated planning, international cooperation, and timely interventions to enhance resilience and mitigate risks in the GoG.

The sea-level rise projections and coastal inundation predictions show that a large portion of the GoG coastline comprises highly populated low-lying deltas, estuaries, and strand-plains that are particularly at risk from rising sea levels^48^ (Fig. 5). Current estimates indicate that 200 million people are at risk of coastal flooding (Alves et al., 2020), and this number is expected to increase as the region experiences some of the fastest population growth rates globally^49^. With more intense and frequent extreme flood events predicted throughout the twenty-first century, the extent of coastal inundation and its associated damages are expected to worsen^50,51^. Regional and annual variability may cause sea-level rise and inundation ranges to exceed predictions in several locations along the GoG. The projected increase in inundated area by 2100 represents a conservative estimate, underscoring the growing coastal flood exposure. In the absence of robust adaptation strategies, the threats to both human populations and cultural heritage along the GoG are escalating rapidly as sea levels rise.

## Discussion and conclusion

This study has identified that among the evaluated models, only eight reliably captured the interannual and seasonal variability across the Tropical Atlantic, specifically in the regions extending from the equatorial band to the GoG. These models consistently mirrored the observed oceanic variability, demonstrating their robustness in representing the complex dynamics of these regions.

A pivotal finding of our research is the persistent one-month lag between the variability observed in the equatorial band and that in the GoG coastal regions. This temporal offset has remained stable over time, underscoring a reliable connection between the equatorial Atlantic and the GoG’s coastal dynamics. This stability is crucial for predicting the propagation of Kelvin CTWs, thereby enhancing our understanding of regional ocean variability. The significance of this time lag as a predictor for CTW propagation is corroborated by Ghomsi, et al.^10^, which reinforces the accuracy of our models in capturing interactions within these oceanic regions.

In addition to rising sea levels, the GoG faces significant risks from extreme events, particularly storm surges. As highlighted by Muis, et al.^50^, storm surges can exacerbate coastal flooding in areas already vulnerable to sea-level rise. These surges, resulting from atmospheric pressure changes and wind effects, can lead to extreme sea levels that exceed normal tidal variations. Consequently, certain areas may become uninhabitable even before they are fully submerged. The anticipated increase in storm intensity due to climate change^52^ further emphasizes the urgency of addressing these impacts, as communities in the GoG are likely to face displacement from severe weather events. This highlights a critical intersection between storm surge impacts and sea-level rise.

Our projections reveal substantial risks for the GoG under different emission scenarios, particularly under the high-emission SSP5-8.5 scenario. This scenario anticipates severe coastal flooding, widespread population displacement by up to 2 million people, and increased 95% inundated area. While these projections are concerning and significant, they are lower than those reported by Kulp and Strauss^20^, who estimated that up to 630 million people globally could be living below the high tide line by 2100 under high emissions, with 72 million at risk in West Africa. Similarly, Vousdoukas, et al.^18^ projected a rise in global coastal flooding exposure from 128 to 171 million people annually in 2010 to 176–287 million by 2100. The comparatively lower displacement figures in our projections are primarily due to our focus on the specific geographical and human livelihood context of the GoG. Furthermore, unlike the authors cited above, we focus on the impact of the current study’s improved sea level projections on coastal flood extents using higher-resolution topography data (~ 30 m), without the additional uncertainties related to extreme sea level events. By incorporating high-resolution data and accounting for the unique features of the region—such as its extensive network of lagoons and estuaries that influence flood dynamics—our sea level rise projections provide a more precise and regionally relevant assessment of local vulnerabilities. Additionally, we did not consider flood defenses in our analysis, which allows us to highlight the unmitigated risks posed by rising sea levels. This approach is critical for understanding the true extent of potential impacts in this region where flood defenses may be limited or non-existent. By presenting a scenario that emphasizes these unmitigated risks, our methodology offers a crucial baseline that reflects the most severe potential outcomes, ensuring that the GoG’s local realities are not overshadowed by broader global trends.

The GoG’s heavy dependence on marine resources, coupled with its critical role in international trade and tourism, heightens the urgency for effective adaptation strategies. Coastal protection infrastructure is vital but must be implemented with caution to avoid adverse impacts on local ecosystems and livelihoods, as highlighted by Duvat and Magnan^53^. Moreover, while managed retreat may be necessary in certain areas, it could lead to the displacement of communities and loss of cultural heritage, as discussed by Ajibade^54^, Petzold and Scheffran^55^.

Our study’s reliability is strongly supported by a series of rigorous validation processes. These include cross-validation with historical data, sensitivity analyses, and direct comparisons with both regional and global models. Notably, our model demonstrated a high degree of accuracy, with the one-month lag showing consistent performance at more than 90% accuracy across the eight selected models. This high level of consistency underlines the robustness of our projections and underscores the credibility of our findings. These methodologies confirm the robustness of our projections and highlight the need for targeted policy interventions.

The findings underscore the critical necessity of enhancing the adaptive capacity of the GoG. Effective adaptation strategies should integrate short-term measures, such as early warning systems and coastal protection infrastructure, with long-term strategies for managed retreat and sustainable land-use planning. Strengthening regional cooperation and fostering knowledge sharing will be crucial for tailoring adaptation efforts to the specific needs and contexts of the GoG.

In light of the escalating impacts of climate change, the experiences and lessons from the GoG offer valuable insights for other vulnerable coastal regions. By mobilizing international support and collaboration for adaptation and resilience-building efforts, we can work towards a more sustainable and equitable future, ensuring that communities and ecosystems are protected against the challenges posed by rising sea levels.

## Data and methods

The gridded monthly SLAs at 1/4- degree spatial resolution from January 1993 to December 2021 from the delayed-time multi-mission (all satellites merged) and the near-real-time datasets distributed by the CMEMS Level 4 (L4) have been used in this study. We subtracted the monthly climatology (calculated for the period 1993 to 2021) from the initial monthly time series to remove the seasonal signal and obtain the interannual anomalies (but note that the procedure does not fully remove the intraseasonal signal). However, Coëtlogon, et al.^56^, Goubanova, et al.^57^ showed that the intraseasonal variability is only influenced by local atmospheric forcing. On the other hand, the interannual variability should be associated with remote equatorial forcing.

We furthermore analyse the Tropical Atlantic Sea surface height outputs of 28 climate models, available in the CMIP6 database obtained from the Climate Data Store^58^. Our methodology involves evaluating CMIP6 models in reproducing interannual variability on a common timescale (1993–2014) with satellite altimetry observations, and later checking among those reproducing this variability whether continuity propagation is also seen. In this region, Ghomsi, et al.^10^ showed a significant correlation (r = 0.68) between the equatorial domain and the 1-degree coastal band domain along longitude, with the anomalies following the same trends throughout the period with lag correlation showing up to one month signal propagation to GoG (see Ghomsi, et al.^10^ for further details). We consider ‘historical’ simulations over the period 1850–2014^59^, and scenario simulations SSP119, SSP126, SSP245 and SSP585 spanning 2015–2100. The new scenarios are based on a matrix using the most common socio-economic pathways (SSPs). Only the first ensemble member of the eight CMIP6 models, ‘r1i1p1f1’ with ocean resolution of 100 km is used for 28 models (see appendix).

To assess the impact of sea level rise along the GoG, we map the projected sea level rise against the coastal topography utilizing the global FabDEM dataset. This dataset is a digital surface model (DSM) at a 30 m resolution that measures the surface of the earth (e.g., removed buildings, trees) providing an improved dataset to calculate the impact of sea level rise along populated centers and vegetated ecosystems^45^. The model reports a mean vertical error ranging from − 0.07 to 0.45 m, varying based on the land cover type. We then calculate the impact of sea level rise from 2023 to 2100 at an annual timestep for three scenarios based on our low, mean, and high uncertainty scenarios for the GoG from the SSP5-8.5 ensemble models. Each calculated sea level rise is used to map the potential inundation extent by only considering the hydrologically connected region (see interactive map in the data availability section). Once the area of each inundation extent has been mapped for each year and for the three scenarios from the SSP5-8.5 ensemble models, we sum the 2020 population distributions within each mapped region based on the WorldPop project at a 100-m resolution^46^.

It is important to note that this methodology has a few shortcomings. Firstly, this approach does not consider flood defenses, and thus represents the increasing sea level risk regionally rather than the actual flood levels for a local region, particularly along river and bay inlets. This methodology is however similar to many previous global storm surge studies meant to highlight the increasing risk and value of existing flood mitigation strategies^20,50^. Secondly, we do not consider population growth or migration patterns as a result of sea level rise, but this provides an accurate baseline with which to compare the potential impact on human livelihoods.

## Electronic supplementary material

Below is the link to the electronic supplementary material.

Supplementary Material 1

## Data Availability

The interactive map showing the scenarios of sea level rise in the Gulf of Guinea can be freely accessed through this link: https://bjornburrnyberg.users.earthengine.app/view/slr-gulf-of-guinea. Population data for the region was sourced from the Grid Population of the World dataset, available at https://sedac.ciesin.columbia.edu/data/collection/gpw-v4. Additionally, the CMIP6 climate projections used in this study were obtained from the Climate Data Store, accessible via https://cds.climate.copernicus.eu/cdsapp#!/dataset/projections-cmip6?tab=overview.
