# Supplementary material for: Sea level rise and coastal flooding risks in the Gulf of Guinea
Source: Sci Rep. 2024 Nov 28;14:29551. doi: 10.1038/s41598-024-80748-w (PMC11605063; doi:10.1038/s41598-024-80748-w)
Supplement: Supplementary file 1 — Supplementary Material 1 [file 41598_2024_80748_MOESM1_ESM.docx]

**SUPPLEMENTARY FIGURES**


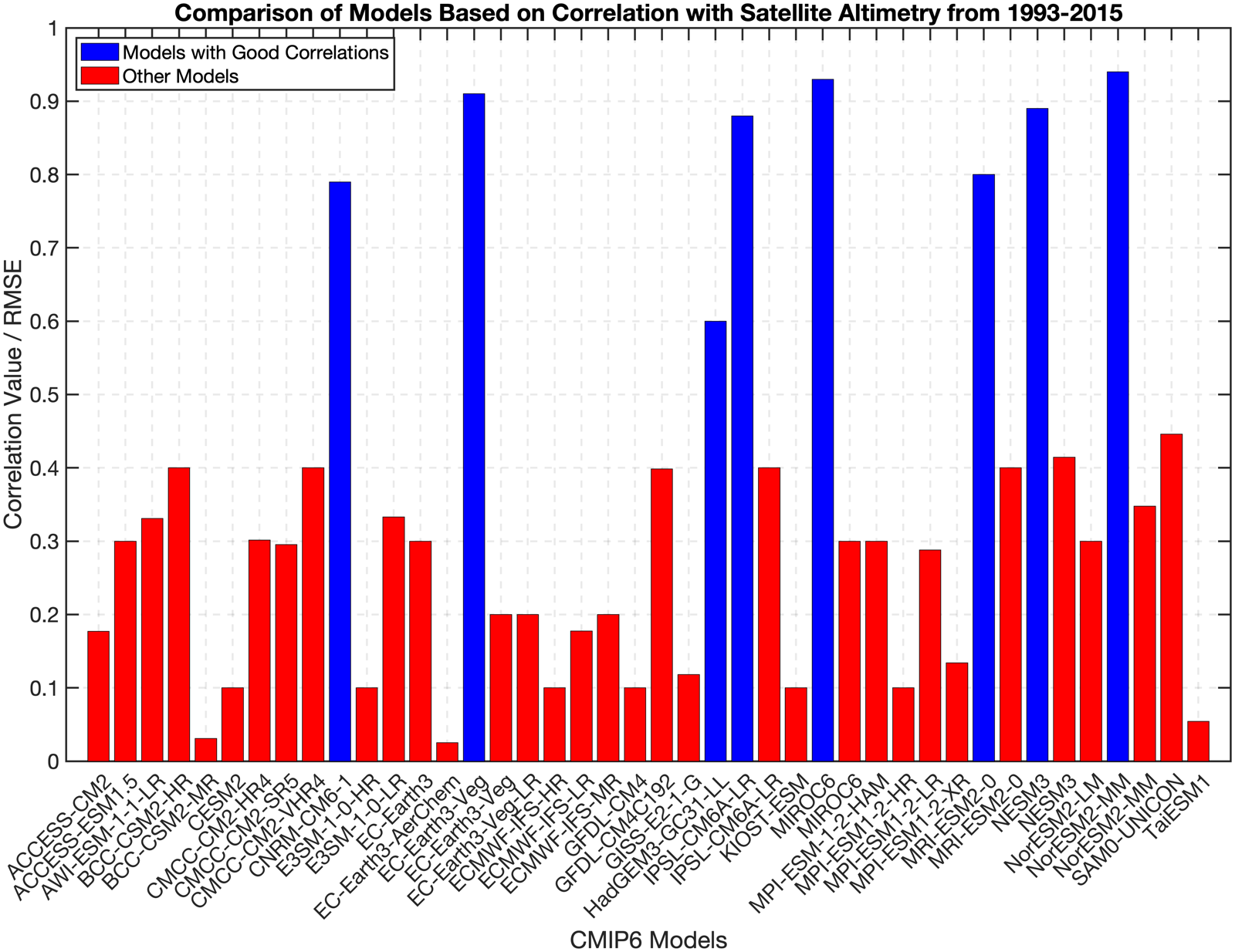


**Figure S1:** *Comparison of the CMIP6 models with SLAs from satellite altimetry.*
